# VEGF-Independent Activation of Müller Cells by the Vitreous from Proliferative Diabetic Retinopathy Patients

**DOI:** 10.3390/ijms22042179

**Published:** 2021-02-22

**Authors:** Sara Rezzola, Jessica Guerra, Adwaid Manu Krishna Chandran, Alessandra Loda, Anna Cancarini, Piergiuseppe Sacristani, Francesco Semeraro, Marco Presta

**Affiliations:** 1 Department of Molecular and Translational Medicine, School of Medicine, University of Brescia, 25123 Brescia, Italy; j.guerra@unibs.it (J.G.); adwaid.krishna@unibs.it (A.M.K.C.); a.loda025@unibs.it (A.L.); 2 Eye Clinic, Department of Neurological and Vision Sciences, University of Brescia, 25123 Brescia, Italy; acancarini@gmail.com (A.C.); piergiuseppe.sacristani@gmail.com (P.S.); francesco.semeraro@unibs.it (F.S.); 3 Italian Consortium for Biotechnology (CIB), Unit of Brescia, 25123 Brescia, Italy

**Keywords:** diabetic retinopathy, inflammation, Müller cells, VEGF, vitreous humor

## Abstract

Proliferative diabetic retinopathy (PDR), a major complication of diabetes mellitus, results from an inflammation-sustained interplay among endothelial cells, neurons, and glia. Even though anti-vascular endothelial growth factor (VEGF) interventions represent the therapeutic option for PDR, they are only partially efficacious. In PDR, Müller cells undergo reactive gliosis, produce inflammatory cytokines/chemokines, and contribute to scar formation and retinal neovascularization. However, the impact of anti-VEGF interventions on Müller cell activation has not been fully elucidated. Here, we show that treatment of MIO-M1 Müller cells with vitreous obtained from PDR patients stimulates cell proliferation and motility, and activates various intracellular signaling pathways. This leads to cytokine/chemokine upregulation, a response that was not mimicked by treatment with recombinant VEGF nor inhibited by the anti-VEGF drug ranibizumab. In contrast, fibroblast growth factor-2 (FGF2) induced a significant overexpression of various cytokines/chemokines in MIO-M1 cells. In addition, the FGF receptor tyrosine kinase inhibitor BGJ398, the pan-FGF trap NSC12, the heparin-binding protein antagonist N-tert-butyloxycarbonyl-Phe-Leu-Phe-Leu-Phe Boc2, and the anti-inflammatory hydrocortisone all inhibited Müller cell activation mediated by PDR vitreous. These findings point to a role for various modulators beside VEGF in Müller cell activation and pave the way to the search for novel therapeutic strategies in PDR.

## 1. Introduction

Proliferative diabetic retinopathy (PDR) is an ocular microvascular complication of diabetes [1]. Currently, it affects more than 93 million people in the world, and it represents the leading cause of acquired blindness in the working age population of industrialized regions [1]. PDR is considered as a multifactorial disease, albeit its pathogenesis is not yet fully understood. Indeed, numerous factors contribute to PDR development, including hyperglycemia, oxidative stress, inflammation, and hypoxia, leading to the damage of the vasculature, as well as of neurons and glial cells in the retina [2,3]. Anti-vascular endothelial growth factor (VEGF) drugs represent the pharmacologic option for PDR treatment to this day [4]. Even though anti-VEGF interventions have shown better outcomes than alternative treatments, limitations to anti-VEGF therapies do exist, including poor response in a significant percentage of patients [5,6,7]. Indeed, VEGF-driven pathways are only part of the complex machinery regulating angiogenesis and inflammation in the eye and the production of other factors may cause resistance to anti-VEGF therapies and limit their efficacy [8]. This creates an unmet need for a better understanding of the pathogenesis of PDR in order to develop more efficacious therapeutic strategies.

Müller cells represent the main glial population of the retina and provide structural support to the neuroretina, radially stretching across its entire thickness. They are the anatomical link between blood vessels and vitreous body, creating a micro-unit involved in the exchange of nourishing molecules and oxygen and in the maintenance of retinal homeostasis [9,10]. During PDR, high blood glucose levels may induce retinal dysfunctions caused by increased levels of oxidative stress, which triggers early neurodegeneration, activation of inflammatory responses, and abnormal neovessel formation [3]. Because of the pathological changes that occur in the retina during PDR, activated Müller cells may undergo reactive gliosis, a non-specific reactive response of glial cells to damage characterized by uncontrolled proliferation, migration, and increased expression of gliosis markers [11,12]. Moreover, Müller cells may undertake a fibrotic trans-differentiation, contributing to the formation of a fibrovascular epiretinal membrane (ERM), which can exert tractional forces on the retinal surface, thus causing retinal detachment [13,14]. In addition, activated Müller cells produce several cytokines and chemokines, contributing to the maintenance of the inflammatory environment, alteration of the blood–retinal barrier (BRB) integrity, and neovascularization [12,15,16].

The vitreous humor undergoes structural and molecular alterations during chronic diabetic conditions that may significantly impact the progression of PDR [3]. Thus, the vitreous obtained via pars plana vitrectomy from patients with PDR can represent a sort of receptacle where pro-angiogenic and proinflammatory mediators with pathological effects on retinal cells accumulate [3,17]. By mimicking the microenvironment facing the diabetic retina, PDR vitreous may therefore represent a valuable tool for a better understanding of the pathogenesis of PDR. Indeed, it has been demonstrated that PDR vitreous induces potent angiogenic and inflammatory responses in endothelial cells [3,18], suitable for the identification of novel pharmacological targets and the evaluation of the efficacy of new drug candidates [3,17,18,19,20,21,22,23].

Here, vitreous fluid obtained from PDR patients was used as a tool to investigate the activation that occurs in Müller cells during PDR. The results show that PDR vitreous induces the acquisition of an activated phenotype in Müller cells, characterized by an increase of cell proliferation and migration, intracellular signaling activation, and proinflammatory cytokine/chemokine expression. Surprisingly, we found that the acquisition of this phenotype is not related to VEGF stimulation, whereas treatment of Müller cells with basic fibroblast growth factor (FGF2), a prototypic member of the FGF family [24], induces a significant increase of the expression of various cytokines/chemokines in MIO-M1 cells. Accordingly, the anti-VEGF drug ranibizumab does not affect Müller cell activation mediated by PDR vitreous whereas treatment with the FGF receptor (FGFR) tyrosine kinase inhibitor BGJ398 [25], the pan-FGF trap NSC12 [26], the multi-target heparin-binding protein antagonist N-tert-butyloxycarbonyl-Phe-Leu-Phe-Leu-Phe Boc2 [20], or the anti-inflammatory drug hydrocortisone inhibits, at least in part, the activity of PDR vitreous samples. Together, these data point to a role for various mediators beside VEGF in the response elicited by PDR vitreous in Müller cells.

## 2. Results

### 2.1. MIO-M1 Müller Cells Are Activated by PDR Vitreous

**Figure 1:**
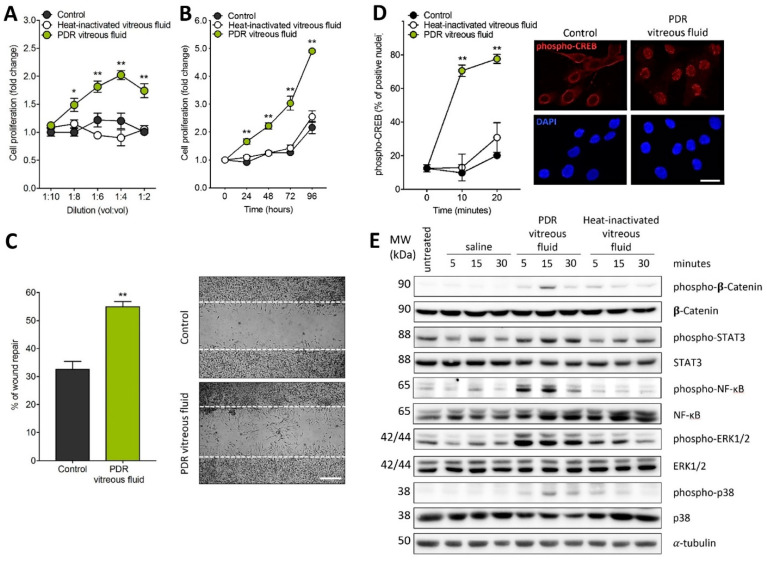
Müller cell activation by proliferative diabetic retinopathy (PDR) vitreous. (**A,B**) MIO-M1 cells were treated with increasing amounts of PDR or heat-inactivated vitreous samples (*vol:vol* dilution in cell culture medium) and counted 72 h thereafter (**A**) or were incubated with 1:4 vitreous dilution and counted at different time points (**B**). Cell proliferation was expressed as fold change in respect to the control. Data are the mean ± SD of 5 independent experiments. * *p* < 0.05 and ** *p* < 0.01 *vs.* control or heat-inactivated vitreous fluid, one-way ANOVA. (**C**) Wounded MIO-M1 monolayers were treated with PDR vitreous fluid. After 24 h, MIO-M1 cells invading the wounded area were quantified by computerized analysis of the digitalized images. Data are the mean ± SD of 2 independent experiments (8 microscopic fields per experimental point). ** *p* < 0.01 *vs.* control, Student’s *t* test. Inset: representative images of the repaired area in control cells (upper panel) and PDR vitreous fluid-treated cells (lower panel). Scale bar = 500 µm. (**D**) MIO-M1 cells were treated with PDR or heat-inactivated vitreous samples. After 0–20 min, the percentage of phospho-CREB immunoreactive MIO-M1 nuclei were quantified (*n* = 80 cells per experimental point). ** *p* < 0.01 *vs.* control or heat-inactivated vitreous, one-way ANOVA. Inset: phospho-CREB immunoreactivity (red) in MIO-M1 cells at 10 min in control (left panels) and after PDR vitreous treatment (right panels); nuclei were stained with DAPI (blue). Data are representative of 2 independent experiments. Scale bar = 25 µm. (**E**) Western blot analysis of the phosphorylation of the signaling proteins β-catenin, STAT3, NF-κB, ERK1/2, and p38 in MIO-M1 cells following 0–30 min of stimulation with PDR or heat-inactivated vitreous samples. Data are representative of 2 independent experiments that gave similar results. MW, molecular weight. Densitometric analysis of the Western blot membranes is shown in Figure S2.

In order to investigate the capacity of PDR vitreous to induce Müller cell activation, MIO-M1 cells were treated with vitreous samples, each pooled from 5–6 diabetic patients. As shown in Figure 1A,B, PDR vitreous stimulates MIO-M1 cell proliferation in a dose- and time-dependent manner. Moreover, it modulates Müller cell motility when assessed in an in vitro wound healing assay (Figure 1C). No significant stimulation of cell proliferation and motility was instead observed when Müller cells were treated with a pool of vitreous samples obtained from patients affected by rhegmatogenous retinal detachment (Figure S1). It must be pointed out that MIO-M1 cells were maintained in culture medium containing 25 mM glucose in these and all the following experiments, thus mimicking more closely a “diabetic-like” microenvironment when cells were treated with PDR vitreous.

In keeping with its capacity to induce Müller cell activation, PDR vitreous induces the rapid nuclear translocation of the proinflammatory transcription factor phospho-cAMP-response element-binding protein (phospho-CREB) (Figure 1D). Accordingly, PDR vitreous rapidly triggers the phosphorylation of a variety of intracellular mediators, including β-catenin, STAT3, ERK1/2, p38, and nuclear factor kappa light chain enhancer of activated B cells (NF-κB) (Figure 1E).

**Figure 2:**
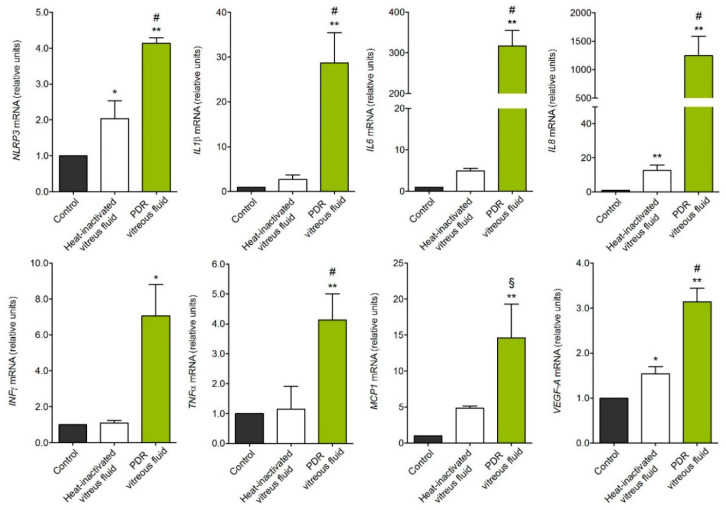
Gene expression analysis of PDR vitreous-activated Müller cells. qPCR analysis of MIO-M1 cells treated with PDR or heat-inactivated vitreous samples. *NLRP3*, *IL1β*, *IL6*, *IL8*, *INFγ*, *MCP1*, and *VEGF-A* expression levels were assessed 4 h after treatment, whereas *TNFα* expression was evaluated at 8 h. Data are representative of 3 independent experiments in triplicate that gave similar results and are expressed as relative units in respect to control. * *p* < 0.05 and ** *p* < 0.01 *vs.* control; § *p* < 0.05 and # *p* < 0.01 *vs.* heat-inactivated vitreous, one-way ANOVA.

In accordance with its capacity to trigger β-catenin and NF-κB activation, treatment with PDR vitreous induces the overexpression of the inflammasome nucleotide-binding oligomerization domain (NOD), leucine-rich repeat (LRR)-containing proteins 3 (*NLRP3*) gene. In parallel, activated MIO-M1 cells upregulate the expression of the inflammatory mediators interleukin 1β (*IL1β*), *IL6*, *IL8*, interferon γ (*INFγ*), monocyte chemoattractant protein 1 (*MCP1*), tumor necrosis factor α (*TNFα*), and *VEGF-A* (Figure 2). No modulation was instead observed for *NLRP1*, *NLRP6*, *CASPASE1*, apoptosis-associated speck like protein (*ASC*), and *IL18* genes, or for *IL4*, *IL17*, and transforming growth factor β (*TGFβ*) (data not shown). In all the assays, negligible activity was exerted by heat-inactivated vitreous, pointing to a proteinaceous nature of the vitreal mediators responsible for the biological activity exerted by PDR vitreous on MIO-M1 cells. However, it is interesting to note that heat-inactivated vitreous retains a significant, even though limited, capacity to stimulate the expression of *NLRP3*, *IL8*, and *VEGF-A* in Müller cells (Figure 2), possibly due to the presence in PDR vitreous of non-proteinaceous components, including bioactive lipids or heat-stable cytokines like TGFβ [3]. Further studies will be required to clarify this point.

### 2.2. PDR Vitreous-Induced Activation of Müller Cells Is Independent from VEGF

**Figure 3:**
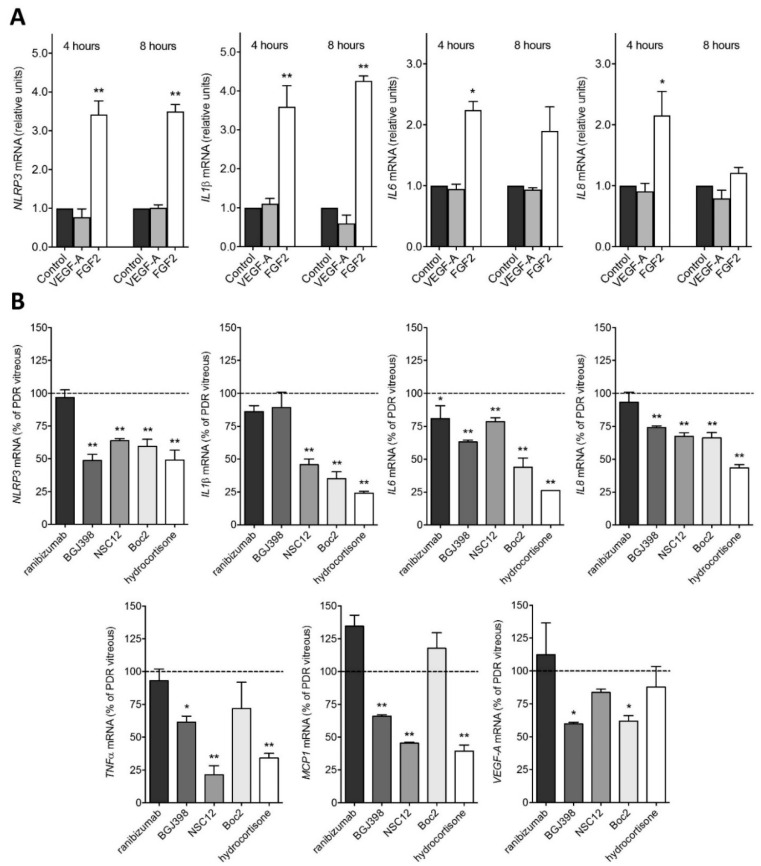
Inhibition of PDR vitreous-induced activation of MIO-M1 cells. (**A**) qPCR analysis of *NLRP3*, *IL1β*, *IL6*, and *IL8* expression in MIO-M1 cells treated with recombinant human VEGF-A or FGF2 for 4 and 8 h. Data are representative of 2 independent experiments in triplicate that gave similar results and are expressed as relative units in respect to control. * *p* < 0.05 and ** *p* < 0.01 *vs.* control or VEGF-A, one-way ANOVA. (**B**) qPCR analysis of *NLRP3*, *IL1β*, *IL6*, *IL8*, *TNFα*, *MCP1*, and *VEGF-A* expression in MIO-M1 cells treated with PDR vitreous for 4 h in the absence or in the presence of 10 µM ranibizumab, 100 nM BGJ398, 10 µM NSC12, 60 µM Boc2, or 10 µM hydrocortisone. Data are representative of 2 independent experiments in triplicate that gave similar results and are expressed as % in respect to PDR vitreous stimulation. * *p* < 0.05 and ** *p* < 0.01 *vs.* PDR vitreous, one-way ANOVA.

The major therapeutic target in PDR is represented by VEGF, which is thought to play a pivotal role in retinal inflammation, vascular leakage, and neovascularization [3]. To evaluate the effect of VEGF stimulation on Müller cells, MIO-M1 cells were treated with recombinant VEGF-A. As shown in Figure 3, VEGF treatment does not modulate the expression of the analyzed proinflammatory genes. These results prompted us to evaluate the effect of the anti-VEGF drug ranibizumab on PDR vitreous-mediated activation of Müller cells. To obtain a complete inhibition of the activity of VEGF, ranibizumab was administered to MIO-M1 cells at a concentration equal to 10 µM, consistent with the dose currently used in clinical practice [27]. In keeping with the results obtained following treatment with recombinant VEGF-A, ranibizumab exerted only a negligible inhibitory effect on the activation of MIO-M1 cells induced by PDR vitreous (Figure 3).

**Figure 4:**
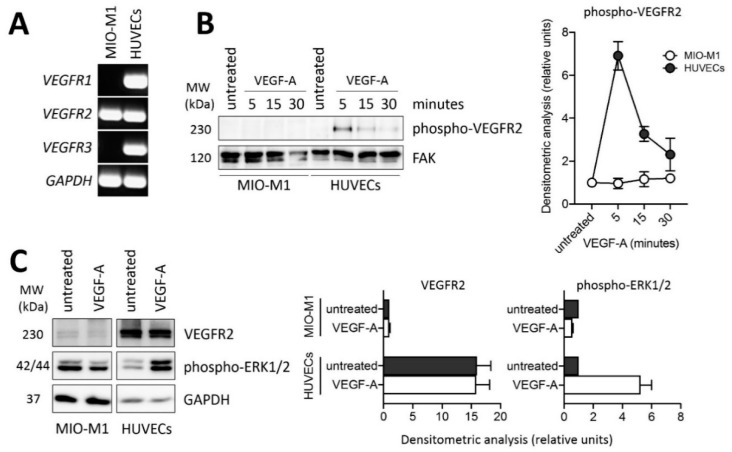
VEGFR2 expression and lack of response in MIO-M1 cells. (**A**) Semi-quantitative RT-PCR analysis of *VEGFR1*, *VEGFR2*, and *VEGFR3* expression in MIO-M1 cells and in human umbilical vein endothelial cells (HUVECs). Data are representative of 2 independent experiments that gave similar results. (**B**) Western blot analysis of VEGFR2 phosphorylation following 0–30 min of stimulation with 30 ng/mL VEGF-A in MIO-M1 cells and HUVECs. Densitometric analysis is shown in the right panel. Data are the mean ± SD of 2 independent experiments. (**C**) Western blot analysis of VEGFR2 protein levels and ERK1/2 phosphorylation following 10 min of stimulation with 30 ng/mL VEGF-A in MIO-M1 cells and HUVECs. Densitometric analysis is shown in the right and far right panel, respectively. Data are the mean ± SD of 2 independent experiments. MW, molecular weight.

Based on these observations, we investigated the expression and activation of VEGFRs in MIO-M1 cells. As shown in Figure 4A, MIO-M1 cells express *VEGFR2* transcripts at levels similar to those detected in human umbilical vein endothelial cells (HUVECs) whereas they fail to express significant levels of *VEGFR1* and *VEGFR3*. However, Western blot analysis of the cell extracts demonstrate that VEGFR2 protein is present at negligible levels in MIO-M1 cells when compared to HUVECs (Figure 4C). Accordingly, VEGF treatment induces a rapid VEGFR2 phosphorylation and ERK1/2 activation in HUVECs, but not in MIO-M1 cells (Figure 4B,C). Together, these data confirm that MIO-M1 cells are irresponsive to VEGF stimulation in our experimental conditions.

FGF2 is the prototypic member of the heparin-binding FGF family [24]. Previous observations showed that FGF2 may induce proliferation and gliotic responses in Müller cells [28]. Accordingly, in contrast with recombinant VEGF-A, recombinant FGF2 induces the overexpression of *NLRP3*, *IL1β*, *IL6*, and *IL8* in MIO-M1 cells, even though to a limited extent when compared to the effect exerted by PDR vitreous (Figure 3). In keeping with these findings, the ATP-competitive tyrosine kinase FGF receptor (FGFR) inhibitor BGJ398 [25] exerts a significant, though partial, inhibition of the overexpression of the proinflammatory genes upregulated by PDR vitreous when administered to MIO-M1 cells at 100 nM, a concentration selective for FGFR1-3 and ineffective for VEGF receptor-2 and various other tyrosine kinase receptors [25] (Figure 3). Similar results were obtained by treating MIO-M1 cells with PDR vitreous in the presence of NSC12, a small FGF-trap molecule which is able to bind and inhibit the activity of all the members of the FGF family [26] (Figure 3). Together, these data raise the hypothesis that stimulation of the FGF/FGFR system triggered by FGF2 and/or by other members of the FGF family may contribute, at least in part, to the activation of Müller cells by PDR vitreous.

Recent observations from our laboratory have shown that the peptide N-tert-butyloxycarbonyl-Phe-Leu-Phe-Leu-Phe (Boc2), widely used as a pan-formyl peptide receptor (FPR) antagonist [29], hampers the angio-inflammatory responses mediated by PDR vitreous on endothelial cells [18]. This FPR-independent effect is due to the capacity of Boc2 to inhibit the binding of a variety of heparin-binding cytokines/growth factors to heparin (including the heparin-binding VEGF-A_165_ isoform, FGF2, connective tissue growth factor, stromal cell-derived factor-1, placenta-derived growth factor-2, high mobility group box-1, platelet-derived growth factor-BB (PDGF-BB), and hepatocyte growth factor), thus preventing their interaction with cell surface heparan-sulphate proteoglycans and cognate receptors [20]. On this basis, in order to assess the effect exerted by a multi-target growth factor/cytokine inhibitor on the activation triggered by PDR vitreous on Müller cells, MIO-M1 cells were incubated with PDR vitreous samples in the absence or in the presence of Boc2. As shown in Figure 3, Boc2 inhibits the upregulation of *NLRP3*, *IL1β*, *IL6*, *IL8*, and *VEGF-A* expression induced by PDR vitreous, whereas it has no effect on the modulation of *TNFα* and *MCP1*.

Various proinflammatory cytokines/chemokines have been detected in PDR vitreous (see [3] and references therein) that are endowed with the capacity to activate Müller cells [30,31,32]. Glucocorticoid receptor signaling exerts an anti-inflammatory action in Müller cells via the modulation of the activity of various transcription factors, including STAT3 and NF-κB (reviewed in [33]). Accordingly, the anti-inflammatory steroid drug hydrocortisone prevented to a significant extent the upregulation of NLRP3, IL1β, IL6, IL8, and VEGF-A that occurs in MIO-M1 cells treated with PDR vitreous (Figure 3). Altogether, these observations point to a role for various vitreal modulators beside VEGF in Müller cell activation during PDR.

## 3. Discussion

The role of retinal glial cells in the pathogenesis of PDR has been thoroughly described [11,12]. In the early stages of DR, Müller cells become hyperactive and start to produce and release angiogenic and neurotrophic factors in order to protect the retina from the insult consequent to the high glucose conditions. However, this response may establish over time an inflammatory milieu that further triggers Müller cell activation and neovascular events typical of the later stages of PDR [11,12,13,14,15,16]. In this frame, the understanding of the reactive responses of Müller cells and of their protective/detrimental effects in PDR is of pivotal importance to bring new therapeutic strategies to patients.

Here, PDR vitreous humor obtained from diabetic patients after pars plana vitrectomy was used as a tool to explore the activation that occurs in Müller cells during PDR. Previous observations have shown that high glucose concentrations may cause the upregulation of various cytokines in Müller cells [31,34,35,36]. In our work, all experiments were performed with MIO-M1 cells maintained in culture medium containing 25 mM glucose, thus mimicking more closely a “diabetic-like” microenvironment when cells were treated with PDR vitreous. The results show that PDR vitreous stimulates MIO-M1 cell proliferation and motility, hallmarks of the gliotic response that characterizes Müller cells [11] and may contribute to ERM formation in PDR patients [37]. No significant stimulation was instead exerted by the vitreous obtained from patients affected by rhegmatogenous retinal detachment, pointing to a specificity of the effect. Accordingly, vitreous samples collected from patients undergoing vitrectomy for diabetic and non-diabetic retinal disorders have shown a different ability to drive the contractile activity of Müller cells, PDR vitreous samples being the most effective (reviewed in [37]). Further studies will be required to assess whether the activation observed in MIO-M1 cells following treatment with PDR vitreous can be induced, and to which extent, by vitreous samples obtained from patients affected by other retinal disorders in which Müller cells may exert a pathogenic role, including macular hole and idiopathic ERM [37,38,39]. Relevant to this point, preliminary observations on a limited set of samples indicate that vitreous from PDR patients with ERM may exert a more potent mitogenic response in MIO-M1 cells when compared to samples from PDR patients without ERM, with no significant difference in their capacity to exert a motogenic stimulus in these cells (data not shown). Analysis of a large cohort of patients will be required to confirm these findings.

Phospho-CREB accumulates in the nucleus of Müller cells in response to acute retinal damage, where it participates in glia de-differentiation, proliferation, and modulation of gene expression [40,41]. Consistent with this observation, treatment with PDR vitreous causes the rapid nuclear translocation of phospho-CREB in MIO-M1 cells. In addition, PDR vitreous induces the phosphorylation of the intracellular mediators β-catenin, STAT3, p38, ERK1/2, and NF-κB, and upregulates the expression of various proinflammatory cytokines/chemokines, including *IL1β*, *IL6*, *IL8*, *INFγ*, *TNFα*, *MCP1*, and *VEGF-A*. This goes in parallel with the upregulation of *NLRP3*, a key component of the NLRP3 inflammasome [42]. These data extend previous observations about the uncontrolled release of IL1β, IL6, and MCP1 by microglial and macroglial cells in diabetic and Akimba animal models [11,43,44,45] and the putative role of NLRP3 inflammasome in PDR [44,46,47].

The activity of the NLRP3 inflammasome is mediated at the transcriptional level (priming) by NF-κB activation and at the post-transcriptional level (activation) by a variety of stimuli (reviewed in [48]). Previous observations have shown that tyrosine kinase signaling might exert both stimulatory and inhibitory effects on the activation of the NLRP3 inflammasome [48,49]. Accordingly, our data demonstrate that FGF2 induces NLRP3 upregulation in Müller cells that express high levels of *FGFR1* and *FGFR2* mRNAs and low levels of *FGFR3* transcript under our experimental conditions (Figure S3B). In addition, the selective FGFR tyrosine kinase inhibitor BGJ398 [25] and the pan-FGF trap NSC12 [26] inhibit *NLRP3* upregulation triggered by PDR vitreous in these cells. A similar inhibitory effect was observed following incubation of PDR vitreous-treated MIO-M1 cells with the pan-heparin-binding protein inhibitor Boc2 or with hydrocortisone. In contrast with FGF2, VEGF does not affect *NRLP3* expression in MIO-M1 cells and the anti-VEGF drug ranibizumab does not prevent the upregulation of *NLRP3* in PDR vitreous-treated MIO-M1 cells. These data point to a role for the FGF/FGFR system and possibly for other mediators, but not VEGF, in NLRP3 inflammasome activation in Müller cells during PDR.

As observed for *NLRP3* expression, our data demonstrate that VEGF-A is unable to induce the upregulation of various proinflammatory cytokines/chemokines in MIO-M1 cells. Accordingly, the anti-VEGF drug ranibizumab does not affect the capacity of PDR vitreous to trigger an inflammatory response in Müller cells when administered at 10 µM, the intravitreal concentration commonly used in the clinical practice [27]. It is worth noticing that similar results were obtained also on endothelial cells, where anti-VEGF drugs showed a limited capacity to hamper the pro-angiogenic/proinflammatory responses induced by PDR vitreous in these cells [18,21]. It must be pointed out that the vitreous samples utilized for our experiments were collected only from PDR patients that received the last drug injection at least 15 days before vitrectomy. Given the intravitreal half-life of anti-VEGF drugs (approx. 5–7 days [50]), the residual levels of the drug in these samples do not affect the activity nor the response of PDR vitreous to anti-VEGF interventions when tested on endothelial cells [19].

No matter the presence of VEGF in PDR vitreous, our results indicate that VEGFR2 protein is present at negligible levels in MIO-M1 cells under our experimental conditions, no VEGFR2 phosphorylation and ERK1/2 activation being observed following VEGF treatment. This occurs despite the levels of *VEGFR2* transcripts being similar to those detected in HUVECs, a prototypic cell type responsive to the VEGF/VEGFR2 axis. This apparent discrepancy may be due to an inefficient translation or instability of the *VEGFR2* transcripts or to an increase in VEGFR2 protein degradation by the ubiquitin/proteasome pathway in MIO-M1 cells when compared to HUVECs. Previous observations had shown the presence of the VEGFR2 protein in Müller cells of rat and murine retina, VEGF neutralization or *Vegfr2* disruption under diabetic conditions leading to Müller cell apoptosis in the two animal models, respectively [51,52]. On the other hand, treatment of MIO-M1 cells with anti-VEGF agents have led to contrasting results with modest or no effect on cell viability/apoptosis [53,54]. Further experiments are required to fully elucidate the role of the VEGF/VEGFR2 system in Müller cells.

A variety of pro-angiogenic/proinflammatory mediators beside VEGF accumulate in the vitreous of PDR patients during disease progression (see [3] and references therein). FGF2 has been detected in ERMs [55,56,57] and pro-inflammatory mediators can induce FGF2 expression in Müller cells [58,59]. Conversely, FGF2 may trigger proliferation and gliotic responses in these cells [28]. Here, we extend these findings by showing that recombinant FGF2 induces the upregulation of proinflammatory genes in MIO-M1 cells. In keeping with this observation, the FGFR tyrosine kinase inhibitor BGJ398 and the pan-FGF trap NSC12 partially inhibit the activation of MIO-M1 cells by PDR vitreous, thus suggesting that the deregulation of the FGF/FGFR system may play a role in Müller cell activation during PDR. FGF2 is the prototypic member of the canonical FGF family that includes the FGF subfamilies FGF1/2/5, FGF3/4/6, FGF7/10/22, FGF8/17/18, and FGF9/16/20 [60] which are able to induce angiogenic, fibrogenic, and inflammatory responses under various pathological conditions [61]. Together, our data indicate that one or more members of the FGF family are present in PDR vitreous and may contribute to its capacity to trigger a proinflammatory response in MIO-M1 cells. Previous observations have shown the capacity of the FGF/FGFR system to activate the canonical WNT/β-catenin pathway via ERK-MAP kinase signaling [62]. In turn, β-catenin may promote NLRP3 inflammasome activation [63]. Further studies will be required to identify the bioactive vitreal member(s) of the FGF family present in PDR vitreous and to fully dissect the FGF/FGFR-dependent signaling leading to the activation of a putative β-catenin/NF-κB/NLRP3 inflammasome pathway in Müller cells.

Finally, the capacity of the multi-target heparin-binding protein antagonist Boc2 and of the anti-inflammatory agent hydrocortisone to inhibit MIO-M1 cell activation triggered by PDR vitreous indicates that other yet unidentified heparin-binding growth factors and inflammatory cytokines may contribute to Müller cell activation. For instance, TGFβ-1 induces glial-to-mesenchymal transition in Müller cells [64], PDGF acts as an autocrine modulator for Müller cells [65,66], and insulin-like growth factor-1 can induce Müller cell proliferation and contractility [65,67,68]. In addition, Müller cells may proliferate in response to the pro-inflammatory cytokine TNFα [32], and IL1β stimulation mediates the upregulation of CCL2, CXCL1, CXCL10, and IL8 chemokines [30,69], as well as the overexpression of IL6 in MIO-M1 cells [31].

Clinical observations demonstrate that anti-VEGF approaches are only partially efficacious for the treatment of PDR patients [5,6,7]. Based on the evidence that anti-VEGF drugs show only a limited effect on the activity exerted by PDR vitreous on Müller cells and endothelial cells [18,21], our results indicate that the characterization of novel drug candidates with different mechanisms of action may contribute, in association with anti-VEGF interventions, to the development of more efficacious therapeutic approaches in PDR.

## 4. Materials and Methods

### 4.1. Reagents

Dulbecco’s modified Eagle medium (DMEM) medium, M199 medium, fetal calf serum (FCS), and SYBR Green PCR master mix were from GIBCO Life Technologies (Grand Island, NY, USA). Endothelial cell growth factor, porcine heparin, penicillin, streptomycin, Triton-X100, BriJ, sodium orthovanadate, protease inhibitor cocktail, 4′,6-diamidino-2-phenylindole (DAPI), hydrocortisone, and anti-α-tubulin antibody were from Sigma-Aldrich (St. Louis, MO, USA). Bradford reagent, enhanced chemiluminescence reagent, and iTaq Universal Syber Green Supermix were from Bio-Rad Laboratories (Hercules, CA, USA). PVP-free polycarbonate filters were obtained from Costar (Cambridge, MA, USA). TRIzol Reagent, Moloney murine leukemia virus (MMLV) reverse transcriptase, anti-phospho-VEGFR2 (pTyr1175), and anti-focal adhesion kinase (FAK) antibodies were from Invitrogen (Carlsbad, CA, USA). ReliaPrep™ RNA Miniprep System was from Promega (Madison, WI, USA). Anti-phospho-NF-κB (pSer311), anti-NF-κB, anti-phospho-STAT3 (pSer727), anti-STAT3, anti-GAPDH, anti-mouse-horseradish peroxidase (HRP), and anti-rabbit-HRP antibodies were from Santa Cruz Biotechnologies (Santa Cruz, CA, USA). Anti-phospho-CREB (pSer133), anti-phospho-β-catenin (pSer552), anti-β-catenin, anti-phospho-ERK1/2 (pThr202/Tyr204), anti-ERK1/2, anti-phospho-p38 (pThr180/pTyr182), anti-p38, and anti-VEGFR2 antibodies were from Cell Signaling Technology (Beverly, MA, USA). Chicken anti-rabbit Alexa Fluor 594 antibody was from Molecular Probes (Eugene, OR, USA). Boc2 was from Phoenix Europe GmbH (Karlsruhe, Germany). Ranibizumab (Lucentis©) was from Novartis (Horsham, UK). BGJ398 was from Selleckchem (Houston, TX, USA). Recombinant human FGF2 was from Tecnogen (Caserta, Italy). Recombinant human VEGF-A (VEGF-A_165_ isoform) was kindly provided by Dr. K. Ballmer-Hofer (PSI, Villigen, Switzerland). Human dermal fibroblast cDNA was kindly provided by Dr. M. Ritelli (University of Brescia, Brescia, Italy). NSC12 was kindly provided by Dr. M. Mor (University of Parma, Parma, Italy).

### 4.2. Human Vitreous Fluid Samples

PDR patients (Table 1) underwent *pars plana* vitrectomy at the Clinics of Ophthalmology (University of Brescia) during the period November 2018–August 2020. Samples were stored at −80 °C. All assays were performed on vitreous samples pooled at random from 5–6 patients. Heat-inactivated vitreous samples were prepared by incubating vitreous pools for 20 min at 95 °C. Pools of vitreous samples obtained from patients affected by rhegmatogenous retinal detachment were used as control.

### 4.3. Cell Cultures

The human Müller cell line Moorfields/Institute of Ophthalmology-Müller 1 (MIO-M1) was obtained from the UCL Institute of Ophthalmology, London, UK [70]. MIO-M1 cells were immediately amplified and stock aliquots were frozen in liquid nitrogen. After thawing, cells were used for no more than 4–5 passages. MIO-M1 cells were grown in DMEM with 4.5 g/L glucose plus 10% FCS and 1.0 mM glutamine. Cells were maintained in a humidified 5% CO_2_ incubator at 37 °C, with medium replaced every 2–3 days until cells reached confluency. Cells were tested regularly for *Mycoplasma* negativity. When compared to human dermal fibroblasts by quantitative PCR (qPCR) analysis, these cells express high levels of the Müller cell markers *vimentin* (*VIM*) and *retinaldehyde binding protein 1* (*RLBP1*, encoding for cellular retinaldehyde binding protein CRALBP) and negligible levels of the *actin α 2* (*ACTA2*) and *S100 calcium-binding protein A4* (*S100A4*) fibroblast markers, encoding for α-smooth muscle actin (α-SMA) and fibroblast-specific protein 1 (S100), respectively (Figure S3A). HUVECs were isolated from human umbilical cords and grown in M199 medium supplemented with 20% FCS, endothelial cell growth factor (100 µg/mL), and porcine heparin (50 µg/mL) as previously described [71].

### 4.4. MIO-M1 Proliferation Assay

MIO-M1 cells were seeded at 5000 cells/cm^2^ in DMEM plus 2.0% FCS. After 3 days, cells were treated with increasing amounts of saline or vitreous diluted in culture medium. After 24, 48, 72, or 96 h, cells were detached with trypsin, suspended in 200 µL of PBS plus 5.0% FCS, and counted with a MACSQuant cytofluorimeter (Milteny Biotec).

### 4.5. MIO-M1 Wound Healing Assay

MIO-M1 cells were seeded at 100,000 cells/cm^2^ in DMEM plus 2.0% FCS. After 3 days, MIO-M1 cell monolayers were scratched with a 200 µL tip to obtain a 2-mm-thick denuded area and cultured in the presence of saline or PDR vitreous diluted 1:4 with culture medium. After 24 h, wounded monolayers were photographed, and the percentage of repaired area was quantified with Fiji software [72].

### 4.6. Western Blot Analysis

MIO-M1 cells were seeded at 50,000 cells/cm^2^ in DMEM *plus* 2.0% FCS. After 3 days of starvation, cells were treated for 0–30 min with saline, 30 ng/mL VEGF or vitreous fluid diluted 1:4 with culture medium. After treatment, cells were lysed in 50 mM Tris–HCl 150 mM NaCl buffer (pH 7.4) containing 1.0% Triton-X100, 0.1% BriJ, 1.0 mM sodium orthovanadate, and protease inhibitor cocktail. Aliquots of each sample containing equal amount of proteins (15–30 μg) were subjected to SDS-PAGE. Gels were transblotted onto a PVDF membrane and blots were blocked with 1.0% BSA for 1 h at room temperature. Western blotting analysis was performed with anti-phospho-β-catenin, anti-β-catenin, anti-phospo-ERK1/2, anti-ERK1/2, anti-phospho-NF-κB, anti-NF-κB, anti-phospho-p38, anti-p38, anti-phospho-STAT3, anti-STAT3, anti-phospho-VEGFR2, anti-VEGFR2, anti-α-tubulin, anti-FAK, or anti-GAPDH antibodies (1:1000). After treating the membranes with appropriate secondary HRP-labeled secondary antibody (1:5000), blots were developed with enhanced chemiluminescence reagent. Images were acquired using a ChemiDoc Touch instrument and band intensity was evaluated with Image Lab 3.0 software (Bio-Rad Laboratories). When specified, MIO-M1 cells were compared to HUVECs for VEGFR2 expression and activation as previously described [73].

### 4.7. RT-PCR Analyses

Semi-quantitative RT-PCR was used to analyze FGFR1-4 and VEGFR1-3 expression in MIO-M1 cells. To this aim, total RNA was isolated from MIO-M1 cells after 3 days of starvation in DMEM plus 2.0% FCS using TRIzol^®^ Reagent according to manufacturers’ instruction. A total of 2.0 µg of total RNA was retro-transcribed with MMLV reverse transcriptase using random hexaprimers in a final 20 μL volume. Then, 1/10th of the reaction was analyzed by semi-quantitative RT-PCR using the primers listed in Table 2. The PCR products were electrophoresed on a 1.5% agarose gel and visualized by ethidium bromide staining. When specified, MIO-M1 cells were compared to HUVECs for VEGFR expression.

For qPCR analysis, MIO-M1 cells were seeded at 50,000 cells/cm^2^ in DMEM plus 2.0% FCS. After 3 days of starvation, cells were treated for 4 h or 8 h with 30 ng/mL VEGF-A, 30 ng/mL FGF2, saline, or vitreous diluted 1:4 with culture medium in the absence or in the presence of 10 µM ranibizumab, 100 nM BGJ398, 10 µM NSC12, 60 µM Boc2, or 10 µM hydrocortisone. Total RNA was isolated using TRIzol^®^ Reagent and ReliaPrep™ RNA Miniprep System according to manufacturers’ instructions. Then, 2.0 µg of total RNA was retrotranscribed and 1/10th of the retrotranscribed cDNA was used for qPCR that was performed with the ViiA 7 Real-Time PCR System (ThermoFisher) using iTaq Universal Syber Green Supermix according to the manufacturer’s instructions. Samples were analyzed in triplicate using the oligonucleotide primers listed in Table 2 and data were normalized to the levels of GAPDH expression.

### 4.8. MIO-M1 Immunofluorescence Analysis

A total of 50,000 cells/cm^2^ was seeded on µ-slide 8-well chambers (Ibidi) in DMEM plus 2.0% FCS. After 3 days of starvation, cells were treated for 0–30 min with saline or vitreous diluted 1:4 with culture medium, fixed in cold methanol, permeabilized with 0.2% Triton-X100, and saturated with 3.0% BSA in PBS (blocking solution). Then, cells were incubated overnight at 4 °C with anti-phospho-CREB antibody (1:800 in blocking solution) and for 1 more hour at room temperature with an anti-rabbit Alexa Fluor 594 secondary antibody (1:500 in blocking solution). Nuclei were counterstained with DAPI and cells were photographed using a Zeiss Axiovert 200M epifluorescence microscope equipped with Apotome and a Plan-Apochromat ×63/1.4 NA oil objective.

### 4.9. Statistical Analysis

Data are expressed as mean ± SD. Statistical significance was evaluated with the GraphPad Prism 7 software (San Diego, CA, USA) using Student’s *t* test or one-way ANOVA followed by Bonferroni multiple comparison post-test to test the probability of significant differences between 2 or more groups of samples, respectively. Differences were considered significant when *p* value < 0.05.

## Figures and Tables

**Table 1:** PDR patients. Data are n unless indicated otherwise and are expressed as mean ± SD.

Patients/Eyes	39/42
**Clinical features**	
**Gender (male/female)**	28/11
**Age (years)**	65 ± 10
**Type 1/type 2 diabetes**	4/35
**Duration of diabetes (years)**	21 ± 6
**Oral hypoglycemic drug treatment**	10/39
**Insulin treatment**	10/39
**Oral hypoglycemic drug + insulin treatment**	19/39
**Glycaemia (mg/dL)**	161 ± 56
**HbA1c (%)**	7.9 ± 1.1
**Neuropathy**	6/39
**Nephropathy**	13/39
**Cardiopathy**	15/39
**Hypertension**	37/39
**Dyslipidemia**	23/39
**Triglycerides (mg/dL)**	120 ± 54
**Cholesterol (mg/dL)**	153 ± 44
**Creatinine (mg/dL)**	1.4 ± 0.7
**Hemoglobin (g/dL)**	13.1 ± 1.6
**Ophthalmic features**	
**PDR**	42/42
**PDR with vitreous hemorrhage**	19/42
**PDR with macular edema**	19/42
**PDR with ERM**	31/38
**Ocular therapies**	
**Intravitreal injection of anti-VEGF blocker**	29/42
**Panretinal laser photocoagulation**	32/42

**Abbreviations:** ERM: epiretinal membranes; PDR: proliferative diabetic retinopathy; VEGF: vascular endothelial growth factor.

**Table 2:** Oligonucleotide primers used for RT-PCR analysis.

Gene	Forward	Reverse
*ACTA2*	5′-AATGGCTCTGGGCTCTGTAA-3′	5′-TTTTGCTCTGTGCTTCGTCA-3′
*FGFR1*	5′-GGGCTGGAATACTGCTACAA-3′	5′-GCCAAAGTCTGCTATCTTCATC-3′
*FGFR2*	5′-GGATAACAACACGCCTCTCTT-3′	5′-GCCCAAAGCAACCTTCTC-3′
*FGFR3*	5′-TGGTGTCCTGTGCCTACC-3′	5′-CCGTTGGTCGTCTTCTTGT-3′
*FGFR4*	5′-AACCGCATTGGAGGCATT-3′	5′-TCTACCAGGCAGGTGTATGT-3′
*GAPDH*	5′-GAAGGTCGGAGTCAACGGATT-3′	5′-TGACGGTGCCATGGAATTTG-3′
*IL1* *β*	5′-GTGGCAATGAGGATGACTTG-3′	5′-GTGGTGGTCGGAGATTCGTA-3′
*IL6*	5′-TGTGTGGGTCTGTTGTAGGG-3′	5′-CCCGTGCAATATCTAGGAAAA-3′
*IL8*	5′-TGTGTGGGTCTGTTGTAGGG-3′	5′-CCCGTGCAATATCTAGGAAAA-3′
*INFγ*	5′-GCAGGTCATTCAGATGTAGCGG-3′	5′-CCACACTCTTTTGGATGCTCTGG-3′
*MCP1*	5′-CTCAGCCAGATGCAATCAA-3′	5′-CACTTCTGCTTGGGGTCA-3′
*NLRP3*	5′-GGACTGAAGCACCTGTTGTGCA-3′	5′-TCCTGAGTCTCCCAAGGCATTC-3′
*RLBP1*	5′-GCTGCTGGAGAATGAGGAAA-3′	5′-TGGTGGATGAAGTGGATGG-3′
*S100A4*	5′-CCTGGATGTGATGGTGTCC-3′	5′-TCGTTGTCCCTGTTGCTGT-3′
*TNFα*	5′-TGCTTGTTCCTCAGCCTCTT-3′	5′-GCTTGTCACTCGGGGTTC-3′
*VEGF-A*	5′-AATCGAGACCCTGGTGGAC-3′	5′-GGTGAGGTTTGATCCGCATA-3′
*VEGFR1*	5′-AGCAGTTCCACCACTTTAGA-3′	5′-GAACTTTCCACAGAGCCCTT-3′
*VEGFR2*	5′-GGAAATGACACTGGAGCCTA-3′	5′-TTTGAAATGGACCCGAGACA-3′
*VEGFR3*	5′-CAACGACCTACAAAGGCTCT-3′	5′-GTAAAACACCTGGCCTCCTC-3′
*VIM*	5′-CGCCAGATGCGTGAAATG-3′	5′-ACCAGAGGGAGTGAATCCAGA-3′

**Abbreviations.***ACTA2*: actin α 2; *FGFR*: fibroblast growth factor receptor; *GAPDH*: glyceraldehyde-3-phosphate dehydrogenase; *IL*: interleukin; *INFγ*: interferon γ; *MCP1*: monocyte chemoattractant protein 1; *NLRP3*: nucleotide-binding oligomerization domain (NOD), leucine-rich repeat (LRR)-containing proteins 3; *RLBP1*: retinaldehyde-binding protein 1; *S100A4*: S100 calcium-binding protein A4; *TNFα*: tumor necrosis factor α; *VEGF-A*: vascular endothelial growth factor-A; *VEGFR*: vascular endothelial growth factor receptor; *VIM*: vimentin.

## Data Availability

The data that support the findings of this study are available from the corresponding authors upon reasonable request.

## Supplementary Materials

The following is available online at https://www.mdpi.com/1422-0067/22/4/2179/s1. Figure S1: Retinal detachment vitreous fluid does not activate MIO-M1 Müller cells; Figure S2: Müller cell signaling activated by PDR vitreous; Figure S3: Molecular characterization of MIO-M1 Müller cells.

## Abbreviations

ACTA2	actin α 2
ERM	epiretinal membrane
FGF2	basic fibroblast growth factor
FGFR	fibroblast growth factor receptor
GAPDH	glyceraldehyde-3-phosphate dehydrogenase
HUVECs	human umbilical vein endothelial cells
IL	interleukin
INFγ	interferon γ
MCP1	monocyte chemoattractant protein 1
NLRP3	nucleotide-binding oligomerization domain (NOD), leucine-rich repeat (LRR)-containing proteins 3
PDGF	platelet derived growth factor
PDR	proliferative diabetic retinopathy
RLBP1	retinaldehyde binding protein 1
S100A4	S100 calcium-binding protein A4
TNFα	tumor necrosis factor α
VEGF-A	vascular endothelial growth factor-A
VEGFR	vascular endothelial growth factor receptor
VIM	vimentin
