# Supplementary material for: VEGF-Independent Activation of Müller Cells by the Vitreous from Proliferative Diabetic Retinopathy Patients
Source: Int J Mol Sci. 2021 Feb 22;22(4):2179. doi: 10.3390/ijms22042179 (PMC7926720; doi:10.3390/ijms22042179)
Supplement: Supplementary file 1 [file ijms-22-02179-s001.pdf]

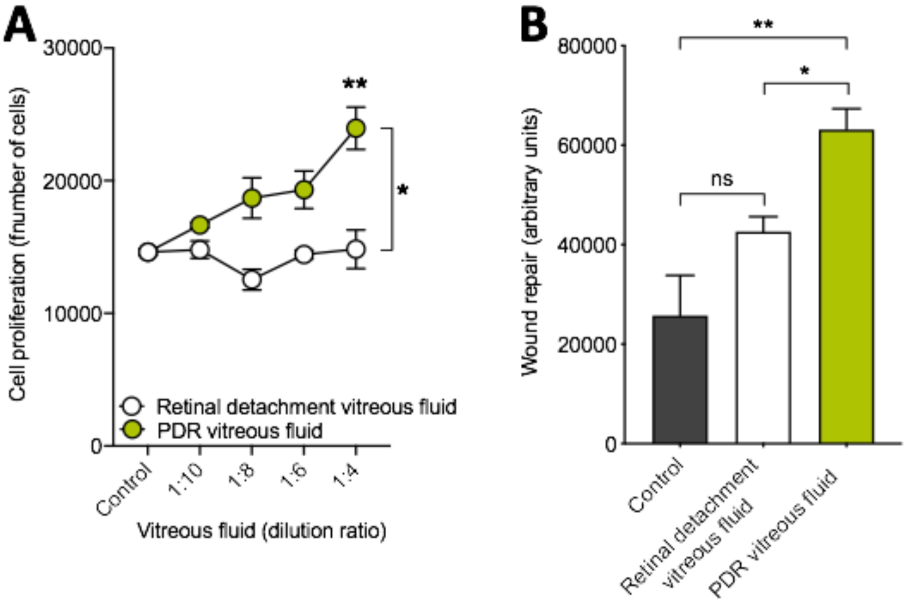

Supplementary Figure 1. Retinal detachment vitreous fluid does not activate MIO-M1 Müller cells. (A) MIO-M1 cells were treated with increasing amounts of PDR or retinal detachment vitreous samples (*vol:vol* dilution in cell culture medium) and counted 72 hours thereafter. Data are the mean ± SD of 2 independent experiments in triplicate. * $p < 0.05$ *vs* retinal detachment vitreous fluid, Student's *t* test, and ** $p < 0.01$ *vs* control, 1-way ANOVA. **(B)** Wounded MIO-M1 monolayers were treated with PDR or retinal detachment vitreous fluid. After 24 hours, MIO-M1 cells invading the wounded area were quantified by computerized analysis of the digitalized images. Data are the mean ± SD of 2 independent experiments (8 microscopic fields per experimental point). * $p < 0.05$ *vs* retinal detachment vitreous fluid, Student's *t* test, and ** $p < 0.01$ *vs* control, 1-way ANOVA; ns, non-significant.

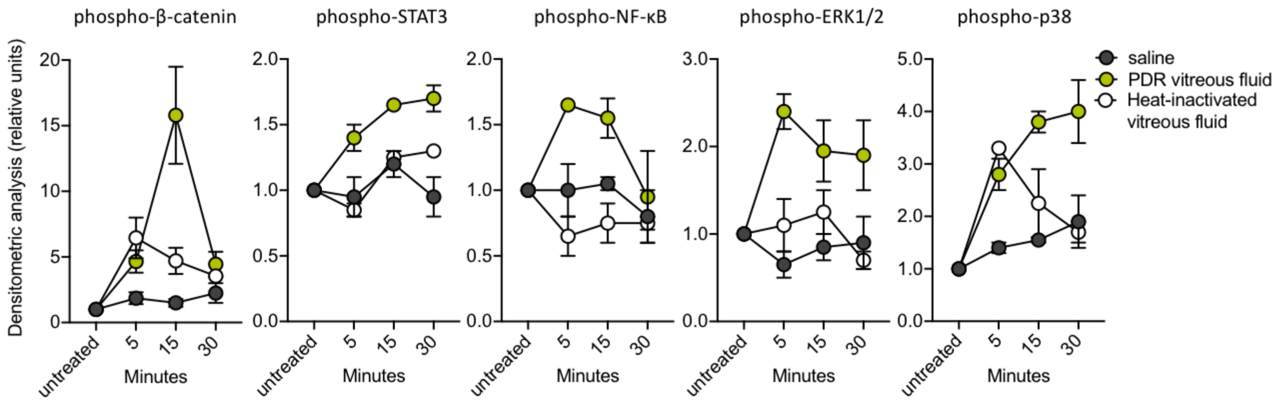

Supplementary Figure 2. Müller cell signaling activated by PDR vitreous. Densitometric analysis of the phosphorylation of the signaling proteins β-catenin, STAT3, NF-κB, ERK1/2, and p38 in MIO-M1 cells following 0-30 minutes of stimulation with PDR or heat-inactivated vitreous samples (see Figure 1E). Data are the mean ± SD of 2 independent experiments.

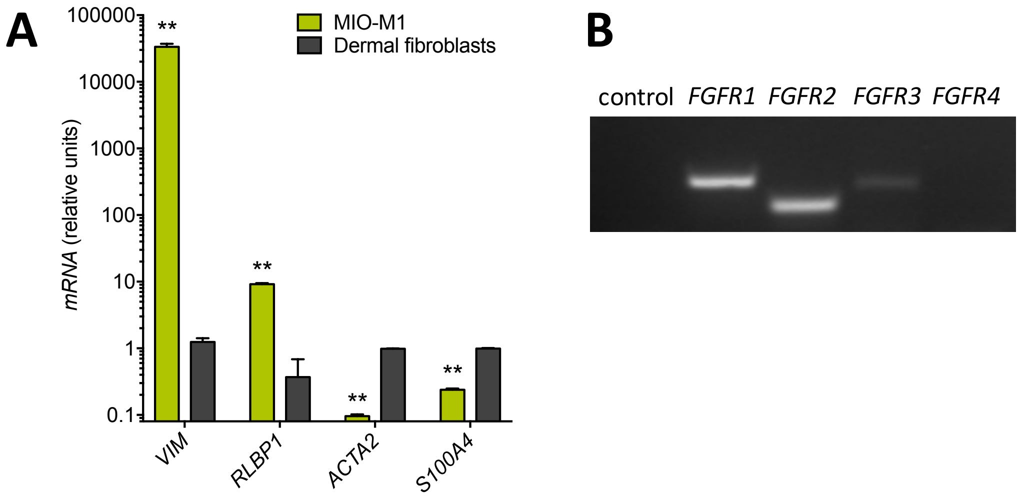

Supplementary Figure 3. Molecular characterization of MIO-M1 Müller cells. (A) qPCR analysis of *VIM*, *RLBP1*, *ACTA2*, and *S100A4* expression in MIO-M1 cells and human dermal fibroblasts. Data are representative of 2 independent experiments in triplicate and are expressed as relative units in respect to *GAPDH* expression. ** $p < 0.01$ *vs* dermal fibroblasts, Student's *t* test. **(B)** Semi-quantitative RT-PCR analysis of *FGFR1*, *FGFR2*, *FGFR3*, and *FGFR4* expression in MIO-M1 cells. Data are representative of 2 independent experiments that gave similar results.
